# Effect of Food Residues on Norovirus Survival on Stainless Steel Surfaces

**DOI:** 10.1371/journal.pone.0021951

**Published:** 2011-08-22

**Authors:** Hajime Takahashi, Ayumi Ohuchi, Satoko Miya, Yukino Izawa, Bon Kimura

**Affiliations:** Department of Food Science and Technology, Faculty of Marine Science, Tokyo University of Marine Science and Technology, Tokyo, Japan; University of Cambridge, United Kingdom

## Abstract

**Background:**

In households and food processing plants, minute food residues left behind from improper cleaning may influence the survivability of human norovirus on surfaces. In this study, the survivability of norovirus on desiccated food residue-attached stainless steel coupons was investigated.

**Methodology/Principal Findings:**

Using murine norovirus-1 (MNV-1) as a surrogate of human norovirus, the survivability of norovirus was investigated on lettuce, cabbage, or ground pork-attached stainless steel coupons. A 6.2 log MPN/ml of MNV-1 infectivity was completely lost at day 30 in residue-free coupons, whereas only a 1.4 log MPN/ml reduction was observed in coupons with residues. Moreover, the disinfective effect of sodium hypochlorite was reduced when residues were present on the coupons.

**Conclusions/Significance:**

This study revealed that the food residues increased the survivability and the resistance to chemicals of norovirus, indicating the need of thorough cleaning in food processing plants and household settings.

## Introduction

Norovirus is a member of the *Caliciviridae* family, and causes gastroenteritis in humans. It is estimated that more than 50% of foodborne outbreaks in the United States may be attributable to norovirus [1]. Likewise, in Japan, this virus accounted for a large portion (about 29%) of the foodborne diseases in 2009 [2]. Besides ingestion of contaminated foods, such as raw oysters, person-to-person transmission can occur, or transmission may occur via contaminated surfaces and aerosols [1]. The infective dose of this virus is very low; even 10 viral particles are sometimes enough to infect an individual [3].

Foodborne outbreaks of norovirus are often associated with infected food handlers in food processing plants [4]–[9]. Therefore, in this study, we investigated the survivability of norovirus on stainless steel, which is a common surface material in food processing plants. Although the efficiency of norovirus attachment to stainless steel surfaces has been studied previously [10], no studies have assessed the survivability of this virus on desiccated surfaces. Notably, the effect of minute food residues on virus survivability, on food-contact surfaces, is of interest in terms of food hygiene in food processing plants and improperly cleaned households. We, therefore, examined the virus survivability by attaching lettuce, cabbage, and pork filtrates on stainless steel coupons. Leafy greens such as lettuce, cabbage, and spinach are increasingly associated with foodborne outbreaks. According to CDC data [11], 502 (4.8%) outbreaks, 18,242 (6.5%) illnesses, and 15 (4.0%) deaths among 10,421 foodborne outbreaks reported during 1973–2006 were caused by leafy greens, and norovirus was responsible for 196 (58.3%) of these outbreaks. In addition, pork was used for a proteinaceous food since food processing plants usually handle many kinds of foods and this kind of food should be investigated for norovirus survivability as well as leafy greens. Moreover, we investigated the effect of sodium hypochlorite on norovirus inactivation on stainless steel surfaces, since this is the preventive measure recommended by the Japanese Ministry of Health, Labour and Welfare.

Although it is imperative that human norovirus is studied in order to prevent further foodborne infections, this virus is uncultivable in the laboratory. Thus, feline calicivirus (FCV) has been widely used as a surrogate in inactivation studies [12]–[15], including inactivation on food-contact surfaces [16]. However, FCV belongs to a different genus, *Vesivirus*, and is known as a respiratory pathogen [17]. Among genus *Norovirus*, porcine norovirus and bovine norovirus are currently unable to be cultured *in vitro*, and moreover, porcine and bovine are difficult to genetically manipulate as animal models [18]. On the other hand, murine norovirus-1 (MNV-1), first isolated from the brain of immunodeficient mouse after intracerebral inoculation in 2003 [19], is now accepted as a surrogate for human norovirus [20]–[22]. MNV-1 is the only norovirus that replicates in cell culture [22], and it is associated with gastrointestinal disease in addition to respiratory infection [19], [22], having low-pH tolerance [21] just like human norovirus [23]. MNV-1 as a model for human norovirus has provided new insight into norovirus lifecycle and interaction with their host. In this study, therefore, we used MNV-1 as a surrogate for human norovirus.

## Results and Discussion

**Figure 1:**
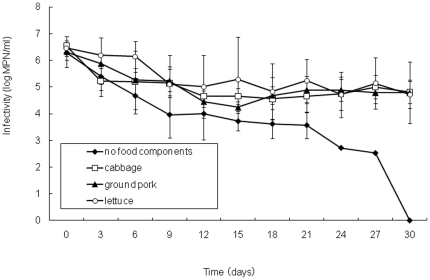
Survival of MNV-1 on stainless steel coupons with or without food residues. Food residue (cabbage, ground pork, or lettuce) -attached stainless steel coupons and coupons without any food component were incubated at 25°C over a 7-day experimental period. Error bars indicate standard deviations obtained from three independent experiments.

We investigated survival and disinfection of human norovirus on food contact surfaces using MNV-1 as an experimental surrogate. First of all, MNV-1 infectivity on stainless steel with or without food residues was investigated (Fig. 1). On the stainless steel coupons without any food residue, MNV-1 infectivity rapidly decreased by more than 2 log MPN/ml, followed by a slow decline and a complete loss at day 30 (the 30-day reduction was significant with *P*<0.05). On the other hand, MNV-1 infectivity on stainless steel coupons with food residues (lettuce, cabbage, and ground pork) decreased by approximately 1.4 log MPN/ml by day 9 and remained at the same level for the rest of the experimental period (the 30-day reduction was not statistically significant with *P*>0.05). Previously better survivability of norovirus in ham than in lettuce and strawberry was reported [24]. The authors ascribed this result to the pH difference of the foods. In our study, we had little difference in norovirus survivability among foodstuffs, possibly because the pH of the food extracts did not differ much from each other (6.89, 6.90, and 6.20 for lettuce, cabbage, and pork, respectively). The difference in norovirus survivability depending on the presence of food residues indicated that the presence of food residues increases the resistance to drying, thus thorough cleanliness in food processing plants is important in preventing foodborne infections. Moreover, if the surfaces were not completely dry, the survivability would be higher [21], suggesting a need for inactivation efforts.

**Figure 2:**
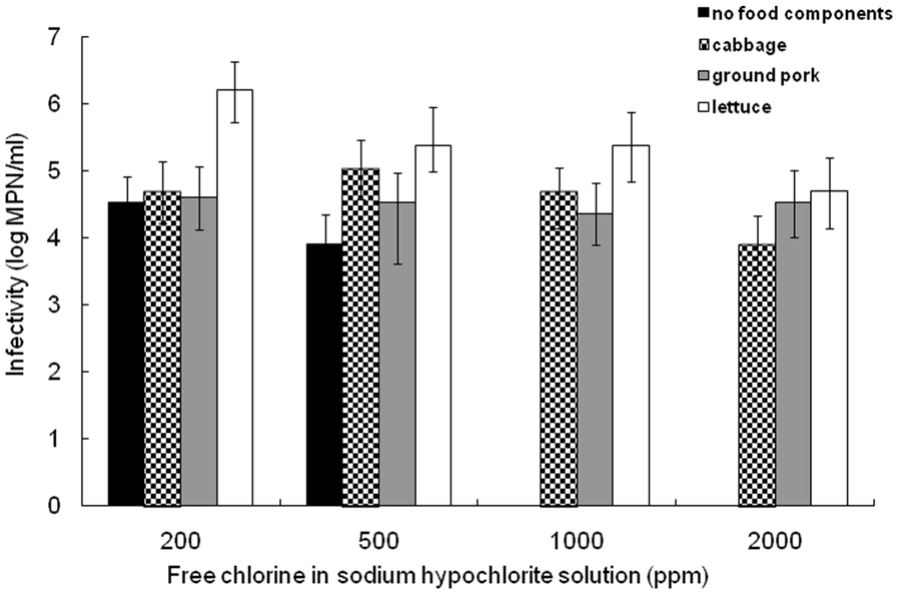
Reduction of MNV-1 on stainless steel coupons by sodium hypochlorite. Stainless steel coupons with or without food residues were exposed for 5 min to various concentration of sodium hypochlorite at room temperature. Error bars indicate standard deviations obtained from three independent experiments.

In Japan, sodium hypochlorite is a government-recommended chemical for norovirus inactivation. The effect of various concentrations of sodium hypochlorite on MNV-1 inactivation is shown in Fig. 2. When no sodium hypochlorite was added to stainless steel coupons with or without food residues as controls, there was no reduction of MNV-1 infectivity (data not shown). Sodium hypochlorite at 1000 ppm was sufficient to inactivate the virus in the absence of food residues. On the other hand, even 2000 ppm had little effect on MNV-1 infectivity on stainless steel coupons with food residues. This is additional evidence of the influence of food components on virus survival. In order to confirm that the virus particles were not washed off but the infectivity was inactivated by the addition of the sodium hypochlorite, real-time reverse transcription PCR was conducted according to the method previously described [25]. The similar RNA level was observed on coupons with or without sodium hypochlorite (data not shown), supporting the inactivation effect of sodium hypochlorite. Girard et al. [10] demonstrated that among major household disinfectants and neutralizers, only sodium hypochlorite was effective against norovirus, and other studies also affirmed the validity of this agent [26], [27]. Our results are also in agreement with these studies only in the absence of food residues. A higher concentration of sodium hypochlorite than is recommended by the government (200 ppm) or the use of other chemical agents should be considered for norovirus inactivation on food contact surfaces in case of the presence of food residues.

Norovirus is often found in fecal material and vomit of infected people, and they are the important sources of spreading viruses. As a consequence, the survivability of the virus in these materials has been previously reported [21]. This study, on the other hand, focused on the survivability on stainless steel which is often used in food processing plants as kitchen utensils and countertop, because the contamination in plants has been often the cause of norovirus outbreak. To our knowledge, this is the first study investigating the effect of food residues on norovirus survivability, which simulates conditions found in food processing plants and households with insufficient cleaning. We found that food residues had a significant effect on norovirus survival, increasing both survivability and chemical resistance. This study reveals that an appropriate and thorough cleaning procedure is imperative to prevent norovirus contamination.

## Materials and Methods

### Survival Study

RAW 264.7 cells were purchased from the American Type Culture Collection (ATCC; Manassas, VA, USA), and maintained as described by Wobus et al. [22]. Murine norovirus strain 1 (MNV-1), kindly provided by Dr. Herbert W. Virgin from Washington University, was inoculated into RAW 264.7 cells and incubated at 37°C in 5% CO_2_ for 72 h. After 3 freeze-thaw cycles, MNV-1 was centrifuged at 8,000× g for 20 min and stored at −80°C until use.

To 100 g of each sample (lettuce, cabbage, or ground pork), an equal amount of sterile phosphate buffered saline (PBS) was added, and the resulting sample was homogenized for 1 min using a Stomacher. The sample was centrifuged (3,500× g for 10 min), and after filtering the supernatant using Advantec filter paper (No. 2, Toyo Roshi, Tokyo, Japan), the sample was filter-sterilized using a membrane filter unit (0.2-µm pore size, Nalgene Labware, Rochester, NY). Stainless steel coupons (2.5×7.5 cm, #400 polish) were autoclaved, washed with distilled water, and sonicated for 1 h, followed by dry-heat sterilization at 180°C for 2 h. The resulting stainless steel coupons were soaked in a sterile food filtrate, quickly removed and then dried on a clean bench. For comparison, stainless steel residue-free coupons were prepared.

Twenty microliters of MNV-1 at 7.9 log MPN/ml (6.2 log MPN) was inoculated onto each of the above-mentioned stainless steel coupons, air-dried and stored up to 30 days inside centrifugation tubes at 25°C. Stainless steel coupons were collected every 3 days. The viral particles were eluted by adding 1 ml of 0.05 M glycine buffer and obtained by scraping the stainless steel coupons with cell scrapers. Viral infectivity was determined by the most probable number method (MPN) described below. MPN was done in triplicate.

### Inactivation by Sodium Hypochlorite

Twenty microliters of MNV-1 at 7.9 log MPN/ml (6.2 log MPN) was inoculated onto each of the above-mentioned stainless steel coupons with or without food residues. After 30-min air-drying, 80 µl of various concentrations (200, 500, 1000, and 2000 ppm) of sodium hypochlorite was added. As negative controls, additional stainless steel coupons with or without food residues were prepared for no sodium hypochlorite addition. After a 5-min exposure, the sodium hypochlorite was neutralized by 0.01 N Na_2_S_2_O_3_ supplemented with 10% fetal bovine serum/Dulbecco's modified Eagle's medium (FBS-DMEM), and MNV-1 was recovered with cell scrapers. The infectivity was determined by MPN method as well. MPN was done in triplicate.

### Determination of Viral Infectivity by the Most Probable Number (MPN) Method

The viral infectivity was determined by MPN method with 95% confidence limits by the 5-tube method. MPN is a statistical tool, deducing the “most probable number” of microorganisms based on the proportion of viral-positive plates to the total number of plates after serial dilutions by factors of ten [28]. This method provides accurate results [28] and is used widely in some fields of microbiological research, including those on norovirus [29], [30]. Briefly, MNV-1 scraped from stainless steel coupons was serially diluted in 10% FBS-DMEM, then inoculated into RAW 264.7 at 2,250,000 cells/well. After a 5-day incubation at 37°C, plates were washed with 100% methanol and the cells were stained with 5% crystal violet. Unstained cells were considered to be dead, which means MNV-1 was present. From the number of MNV-1-positive wells among five wells at the appropriate dilution, the MPN was determined based on a conversion table [31].

### Statistical Analysis

For comparisons on virus survivability among treatments, statistical analysis was performed using the one-way analysis of variance with Tukey's multiple comparison test (SPSS version 17.0, SPSS Japan Inc., Tokyo, Japan).

## References

[1] Centers for Disease Control and Prevention (2010). Norovirus: Technical Fact Sheet.. http://www.cdc.gov/ncidod/dvrd/revb/gastro/norovirus-factsheet.htm.

[2] Ministry of Health, Labour and Welfare (2009). Food Poisoning Surveillance Report.. http://www.mhlw.go.jp/topics/syokuchu/04.html#4-2.

[3] Moe C, Sobsey MD, Stewart P, Crawford-Brown D (1999). Estimating the risk of human calicivirus infection from drinking water..

[4] Friedman DS, Heisey-Grove D, Argyros F, Berl E, Nsubuga J (2005). An outbreak of norovirus gastroenteritis associated with wedding cakes.. Epidemiol Infect.

[5] Kuritsky JN, Osterholm MT, Greenberg HB, Korlath JA, Godes JR (1984). Norwalk gastroenteritis: a community outbreak associated with bakery product consumption.. Ann Intern Med.

[6] Malek M, Barzilay E, Kramer A, Camp B, Jaykus LA (2009). Outbreak of norovirus infection among river rafters associated with packaged delicatessen meat, Grand Canyon, (2005).. Clin Infect Dis.

[7] Parashar UD, Dow L, Fankhauser RL, Humphrey CD, Miller J (1998). An outbreak of viral gastroenteritis associated with consumption of sandwiches: implications for the control of transmission by food handlers.. Epidemiol Infect.

[8] Patterson W, Haswell P, Fryers PT, Green J (1997). Outbreak of small round structured virus gastroenteritis arose after kitchen assistant vomited.. Commun Dis Rep.

[9] Widdowson MA, Sulka A, Bulens SN, Beard RS, Chaves SS (2005). Norovirus and foodborne disease, United States, 1991–2000.. Emerg Infect Dis.

[10] Girard M, Ngazoa S, Mattison K, Jean J (2010). Attachment of noroviruses to stainless steel and their inactivation, using household disinfectants.. J Food Prot.

[11] Herman K, Ayers T, Lynch M (2008). Foodborne disease outbreaks associated with leafy greens, 1973–2006..

[12] Doultree JC, Druce JD, Birch CJ, Bowden DS, Marshall JA (1999). Inactivation of feline calicivirus, a Norwalk virus surrogate.. J Hosp Infect.

[13] Duizer E, Bijkerk P, Rockx B, de Groot A, Twisk F (2004). Inactivation of caliciviruses.. Appl Environ Microbiol.

[14] Urakami H, Ikarashi K, Okamoto K, Abe Y, Ikarashi T (2007). Chlorine sensitivity of feline calicivirus, a norovirus surrogate.. Appl Environ Microbiol.

[15] Whitehead K, McCue KA (2010). Virucidal efficacy of disinfectant actives against feline calicivirus, a surrogate for norovirus, in a short contact time.. Am J Infect Cont.

[16] Gulati BR, Allwood PB, Hedberg CW, Goyal SM (2001). Efficacy of commonly used disinfectants for the inactivation of calicivirus on strawberry, lettuce, and a food-contact surface.. J Food Prot.

[17] Hoover EA, Kahn DE (1975). Experimentally induced feline calicivirus infection: clinical signs and lesions.. J Am Vet Med Assoc.

[18] Wobus CE, Thackray LB, Virgin HW (2006). Murine norovirus: a model system to study norovirus biology and pathogenesis.. J Virol.

[19] Karst SM, Wobus CE, Lay M, Davidson J, Virgin HW (2003). STAT1-dependent innate immunity to a Norwalk-like virus.. Science.

[20] Bae J, Schwab KJ (2008). Evaluation of murine norovirus, feline calicivirus, poliovirus, and MS2 as surrogates for human norovirus in a model of viral persistence in surface water and groundwater.. Appl Environ Microbiol.

[21] Cannon JL, Papafragkou E, Park GW, Osborne J, Jaykus LA (2006). Surrogates for the study of norovirus stability and inactivation in the environment: a comparison of murine norovirus and feline calicivirus.. J Food Prot.

[22] Wobus CE, Karst SM, Thackray LB, Chang KO, Sosnovtsev SV (2004). Replication of norovirus in cell culture reveals a tropism for dendritic cells and macrophages.. PLoS Biol.

[23] Graham DY, Jiang X, Tanaka T, Opekun AR, Madore HP (1994). Norwalk virus infection of volunteers: new insights based on improved assays.. J Infect Dis.

[24] Mattison K, Karthikeyan K, Abebe M, Malik N, Sattar SA (2007). Survival of calicivirus in foods and on surfaces: experiments with feline calicivirus as a surrogate for norovirus.. J Food Prot.

[25] Stals A, Baert L, Botteldoorn N, Werbrouck H, Herman L (2009). Multiplex real-time RT-PCR for simultaneous detection of GI/GII noroviruses and murine norovirus 1.. J Virol Metthods.

[26] Belliot G, Lavaux A, Souihel D, Agnello D, Pothier P (2008). Use of murine norovirus as a surrogate to evaluate resistance of human norovirus to disinfectants.. Appl Environ Microbiol.

[27] Park GW, Boston DM, Kase JA, Sampson MN, Sobsey MD (2007). Evaluation of liquid- and fog-based application of Sterilox hypochlorous acid solution for surface inactivation of human norovirus.. Appl Environ Microbiol.

[28] Entis P, Entis P, Entis M (2002). Most probable number (MPN).. Food microbiology: the laboratory.

[29] Jimenez L, Chiang M (2006). Virucidal activity of a quaternary ammonium compound disinfectant against feline calicivirus: A surrogate for norovirus.. Am J Infect Control.

[30] Katayama H, Haramoto E, Oguma K, Yamashita H, Tajima A (2008). One-year monthly quantitative survey of noroviruses, enteroviruses, and adenoviruses in wastewater collected from six plants in Japan.. Water Res.

[31] Garthright WE, Jackson GJ (1995). Appendix 2. Most probable number from serial dilutions, p.2.08.. FDA bacteriological analytical manual.

